# Nutritional Status and Frailty Improvement through Senior-Friendly Diet among Community-Dwelling Older Adults in South Korea

**DOI:** 10.3390/nu15061381

**Published:** 2023-03-13

**Authors:** Hye-Ri Shin, Young-Sun Kim, Yoo-Kyung Park, Seul-Ki Koo, Woo-Hyun Son, Jae-Won Han, Eun-Ha Son, Hae-Jin Kang, Kyeong-Hee Choi, Jin-Soo Han, Hyun-Sun Lee, Hee-Sook Lim

**Affiliations:** 1 Department of Gerontology, AgeTech-Service Convergence Major, Graduate School of East-West Medical Science, Kyung Hee University, Yongin 17104, Republic of Korea; ltc.shinhyeri@gmail.com (H.-R.S.); ysunkim@khu.ac.kr (Y.-S.K.);; 2 Department of Medical Nutrition, AgeTech-Service Convergence Major, Graduate School of East-West Medical Science, Kyung Hee University, Yongin 17104, Republic of Korea; ypark@khu.ac.kr (Y.-K.P.);; 3 The Food Industry Promotional Agency of Korea, Iksan 54576, Republic of Korea

**Keywords:** health services for the aged, senior-friendly food, nutritive values, frailty, physical function

## Abstract

Considering that Korea’s aging population is rapidly increasing, health serves as an indicator of older adults’ quality of life, and dietary life directly affects their health. For health maintenance and improvement, preventive healthcare measures including safe food selection and nutritional supply are needed. This study aimed to evaluate the effect of senior-friendly diet on nutrition and health status improvement in older adults receiving community care. A total of 180 older adults were analyzed, with 154 and 26 in the senior-friendly diet intervention group and the general diet group, respectively. Surveys, blood tests, and frailty evaluations were conducted before and after the study. After 5 months of intervention, the blood status, nutrient intake, and frailty level were evaluated. The participants’ mean age was 82.7 years, and 89.4% of them were living alone. In both groups, energy, protein, vitamin A, vitamin D, vitamin C, calcium, and magnesium intake were insufficient initially but generally improved after the intervention. Especially in the intervention group, energy, protein, vitamin D, vitamin C, and folic acid intake significantly increased. The frailty level also slightly improved, and the malnutrition rate was reduced. Even after the passage of time, the improvement effect size significantly differed between the groups. Therefore, resolving and supporting meals corresponding to the physiological needs of the older adults has a great impact on improving their quality of life, and such special consideration is a reasonable way to respond to a super-aged society.

## 1. Introduction

Owing to the improvement of living quality and the development of medical technology as a result of economic growth, the average life expectancy in South Korea has increased; thus, population aging is becoming more pronounced [1]. In 2020, approximately 16% of the total population in Korea was aged 65 years or older, and this percentage is estimated to increase to 21% by 2025, thereby entering a super-aged society [2]. As one grows older, their activity level and basal metabolic rate decreases [3]. Masticatory-swallowing and digestive abilities also decline, so food intake decreases and the burden of eating increases [4,5]. Furthermore, grocery shopping and meal preparation have become difficult, leading to malnutrition and nutritional imbalance [4,5]. Poor dietary management in old age leads to various diseases, including lifestyle-related diseases and simple malnutrition, resulting in weakness and eventually, a significant decrease in the quality of life [6]. According to the 2014 Survey on the Elderly results in Korea, approximately half of the older adults in Korea had an “adequate level” of nutritional management; however, 29% required attention in nutrition management, and 20% required improvement in nutrition management [7]. Therefore, managing older adults’ diets in ways that are suitable for a good level of self-reliance and health is crucial to help older adults lead a healthy daily life.

Accordingly, various policy studies and implementations are being conducted to promote proper welfare for older adults in Korea. “Community integrated care” (community care) is a project implemented by the Ministry of Health and Welfare in June 2019, and is a Korean-style service that provides integrated services such as housing, health care, nursing care, and care according to the individual needs of adults aging in place [8]. As part of the community care project, meal delivery services are provided for older adults at home through the “Elderly Health Function Recovery Support Project” and “Customized Nutritious Food Support Project.” In the Welfare of the Elderly Act, the “older adults at home” is defined as an older adult leading a daily life at home [8,9]. Depending on the degree of independent living and health level, these older adults are classified into those who can live independently, those who can live independently but are not healthy, those who cannot live independently and have difficulty moving, and those who cannot live independently and live in long-term care facilities [10]. Usually, meal delivery services are mainly for older adults at home who cannot live independently but are not healthy and those who cannot live independently and have difficulty moving around [11]. Meanwhile, according to the community care project, nutritional support programs are being implemented for older adults at home by local governments in South Korea, but meal providers rarely identify the nutritional status of the target and reflect it in program operation. To increase the effectiveness of the nutritional support program, healthcare providers need to evaluate the nutritional status of older adults first, develop and promote programs that select individuals according to the evaluated nutritional status, and provide customized support for each individual.

In Korea, the Ministry of Agriculture, Food, and Rural Affairs established an age-friendly industry standard in December 2017 which has been used as a voluntary labeling system for food industries [12]. Furthermore, Korea’s senior-friendly food standard (KS H 4897) was introduced, and is divided into stages based on physical properties; it also includes minimum quality standards for nutritional components in consideration of nutritional imbalance, which is common among older adults [13]. The size of Korea’s senior-friendly food industry ranks second among all industries, and is reportedly 17.6 billion won in 20 years [14]. Although the standard policy for senior-friendly food is being continuously expanded, conducting practical field application evaluations and empirical studies is necessary to secure scientific basis data to revitalize the industry and enhance competitiveness [15]. Therefore, this study aimed to scientifically verify whether nutritional intake and clinical indicators were affected by the development of a diet in which senior-friendly foods were provided to older adults.

## 2. Materials and Methods

### 2.1. Participants

This study targeted older adults living at home who were receiving community care. Considering the lack of an identical model at home and abroad, we reviewed the most similar model and calculated the number of participants [16]. The rate of change from malnutrition to normal through malnutrition management was approximately 30%, and the conversion rate from malnutrition to normal was assumed when nutrition was not managed. The ratio of the intervention and control groups was assumed to be 5 according to a type I error of 5% and a power of 90%. The calculated final sample size was 150 in the intervention group and 30 in the control group; when a dropout rate of 9% was applied, the size was 165 and 33, respectively. The inclusion criteria were adults aged 65 years or older and those who agreed with the researcher and their guardians. Those with impaired oral intake, cognitive impairment (e.g., dementia), and nonpermanent residence were excluded. We initially recruited 204 participants and randomly assigned them to the intervention and control groups. At the end of the study, 24 were excluded because of survey refusal (*n* = 20), death (*n* = 1), and hospitalizations (*n* = 3). Thus, the study ended with a total of 180 participants (intervention group: 154, control group: 26).

### 2.2. Meal Provision

Our participants were vulnerable; thus, they were cared for and given lunch boxes every day. Conventional lunch boxes have been provided with nutritious values suitable for older adults. Accordingly, the control group was given the existing meal, while the intervention group was given a meal consisting of senior-friendly foods. Senior-friendly foods in Korea refer to foods manufactured/processed by adjusting their physical properties for easy digestion or by adjusting its nutritional content to help the older adults eat or digest food [17,18]. With the enactment of the Aging-Friendly Industry Promotion Act, products with high convenience and safety in senior-friendly foods are designated as excellent foods [18,19]. To balance the diet of only two groups, we replaced the senior-friendly foods with applicable dishes and reviewed food groups and nutritional values to ensure no significant differences. Meals were provided to all participants for 5 months, and a total of 64 senior-friendly foods were applied to the diet.

### 2.3. Effective Indicators

The primary endpoints used to verify effectiveness were frailty and blood parameters, and the secondary endpoints were nutritional status and nutrient intake status. Frailty can be evaluated in various ways, but a method of evaluating it through a physical function test is recently recommended, that is, the Short Physical Performance Battery (SPPB) [20,21]. SPPB evaluates physical function, frailty, sarcopenia, and fall risk in various clinical situations and predicts health risks that may occur in the future. In particular, it measures the static balance test in three postures, the gait speed test, and the time to get up from a chair five times [22]. In this study, an electronic SPPB (eSPPB) meter was used. The total score is 12 points; a score of 6 or less was considered disability and frailty, and a score of 7–9 was considered prefrailty [20,21].

The participants’ systolic blood pressure, diastolic blood pressure, and grip strength were measured twice using a sphygmomanometer and a grip dynamometer; then, the average value was calculated. Blood was also collected to check the levels of blood glucose, c-reactive protein, total cholesterol, low-density lipoprotein cholesterol (LDL-C), high-density lipoprotein cholesterol (HDL-C), triglyceride, hemoglobin, and hematocrit through an analyzer. Blood pressure, glucose, lipid status, and anemia status were then evaluated.

The nutritional status was investigated using the Mini Nutritional Assessment (MNA^®^) and evaluated as either a good nutritional status, at risk for malnutrition, or malnutrition [23]. The nutrient intake status was evaluated using CAN PRO 5.0 (Computer Aided Nutritional Analysis Program for Professionals, The Korean Nutrition Society, Seoul, Korea) after examining food intake for 2 days using the 24 h recall method. In addition, age, sex, activity level, family status, disease history, oral condition, swallowing condition, quality of life, and body mass index (BMI) were measured as general variables. We used the oral health impact index (OHIP-14) tool for the oral condition; the total response score is between 14 and 17 points [24]. The higher the total score, the lower the oral-health-related quality of life. For the swallowing status, we used dysphagia handicap index (DHI), and a total of 25 items were surveyed [25]. The higher the score, the more severe the swallowing disorder. All indicators were compared and evaluated before and after the intervention.

### 2.4. Nutritional Education Program

Ten nutrition education programs were conducted for the participants’ smooth research progress and investigation, promotion of compliance, and dietary life management. A nutrition expert visited the participants’ house twice a month and provided 1:1 personal nutrition education and counseling. The program mainly consists of topics such as dietary guidelines for the older adults, nutritional management methods for geriatric diseases, introduction of senior-friendly foods, food purchasing, and hygienic and safe meals. Both the control and intervention groups were equally conducted.

### 2.5. Statistics Analysis

We used linear mixed models for endpoint indicators (MNA, energy, protein, eSPPB, hand grip, quality of life, blood pressure, cholesterol, triglyceride, hemoglobin, and hematocrit) to perform between-group comparisons by an intention-to-treat approach. The linear mixed model included group, time, and group-by-time interaction. Each group’s data presented changes from baseline to 5 months, and they were determined by the model’s time-by-group interaction coefficients. Comparisons of the intervention and control groups were analyzed using *t*-test for continuous data and χ^2^ for categorical data. All comparisons were two-sided, and *p* < 0.05 was considered statistically significant. All statistical data were analyzed using the STATA 17.0 software.

## 3. Results

### 3.1. General Characteristics of the Participants

The mean age was 82.7 years, and those over 85 years old accounted for 40.6% (Table 1). Females numbered more than males (62.8%). As for the active state, the rate of independent living was 88.3%, and single-person households accounted for 89.4%. Regarding disease history, cardiovascular diseases such as hypertension and dyslipidemia were the highest (66.7%), and the polypharmacy rate (≥3 drugs) was 80.6%, as observed in most of the participants with chronic diseases. The mean BMI was 23.5 kg/m^2^. Before the study, the baseline characteristics were not significantly different between the two groups.

### 3.2. Nutritional Status and Dietary Quality

**Figure 1:**
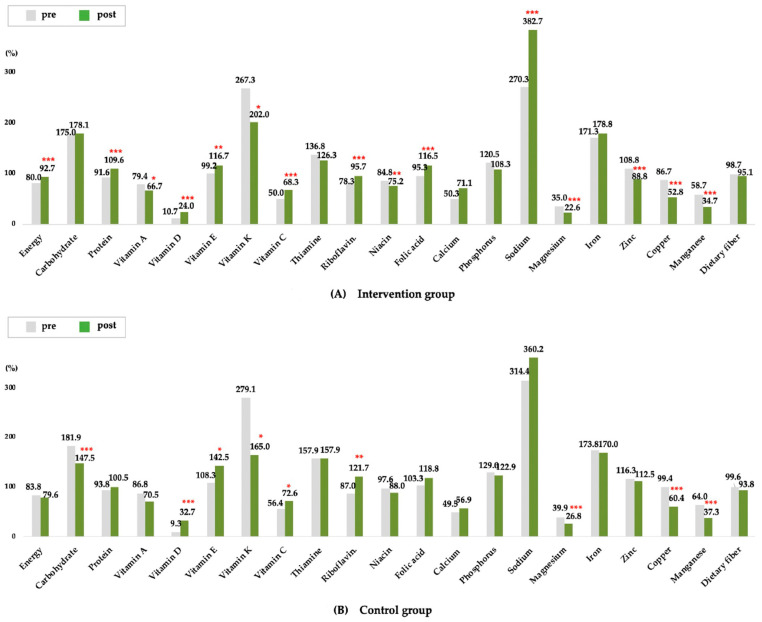
The ratio of intake to the appropriate nutrient standard for each group. Data are reported as dietary reference intakes versus actual intake (%) for the elderly in Korea. Asterisks indicate significant differences pre-post results (* *p* < 0.05, ** *p* < 0.01, and *** *p* < 0.001).

Table 2 and Figure 1 shows the results of analyzing the nutritional status and nutrient intake. In assessing nutritional status through MNA, the proportion of malnutrition (risk of malnutrition and malnutrition) decreased from 53.9% to 50.0% in the control group, but this result was not significant. In the intervention group, it was significantly reduced from 52.6% to 42.9%. As a result of nutrient intake analysis, the intervention group’s calorie intake increased by about 200 kcal and protein by 10 g, and most nutrient intake tended to increase as the overall meal amount increased. In the control group, there was no change in calories and protein, but carbohydrates decreased, and fat intake increased. Figure 1 shows the nutrient recommendation value as 100% and the percentage of satisfaction compared to the standard as a graph. Energy intake in the intervention group increased from 80% to 92.7% compared with the recommended amount for older adults in the Korean nutrient intake standards. In addition, protein exceeded 100%, and the intake generally increased after the intervention period. Conversely, nutrients that remained less than 100% were vitamin A, vitamin D, vitamin C, niacin, calcium, and magnesium. The control group’s intake was at a more vulnerable level, and energy was lowered from 83.8% to 79.6%.

### 3.3. Improving Physical Function and Blood Test

The baseline results of eSPPB were not significantly different between the intervention and control groups, and the mean total score indicated frailty. Specifically, physical function tended to decrease over time in the control group, and the frailty and prefrailty ratios significantly improved in the intervention group. In the blood test, the total cholesterol and triglyceride levels of the intervention group significantly decresed, while the hemoglobin level of the control group significantly decreased (Table 3).

### 3.4. Analysis of Differences in Indicators for Each Group

Table 4 shows the results of analyzing the significant differences between groups using the double-difference analysis method. The nutritional status evaluation score, energy intake, protein intake, eSPPB score, grip strength, hemoglobin, and hematocrit were the indices showing significant differences between the groups. In the control group, most of the indicators tended to decline over time, whereas in the intervention group, the nutritional status and functional health status improved. However, hemoglobin and hematocrit decreased in both groups, but the decrease in the control group was significantly greater.

## 4. Discussion

South Korea ranks first in the world in the rate of aging, with a life expectancy of 86.6 years among Korean females [26]. In this study, the mean age of our participants was 82.7 years, and 40.6% of them were aged 85 years or older, indicating a high level. In addition, females accounted for 62.8%. Thus, Korea’s actual population aging rate reached a very high level compared with the data obtained in this study. Furthermore, single-person households accounted for 89.4%, which is approximately 4.5 times higher than the 19.8% in the 2020 Korea Elderly Survey [27]. Our study population had a large proportion of older adults living alone, suggesting that the proportion of those living alone in South Korea is generally higher than the proportion of those living with other household members. In the 2020 National Health Statistics, more than half of the older adults aged 65 years or older had hypertension, and dyslipidemia accounted for 65.2% and 75.0% [28]. In addition, more than 80.6% of our participants took three or more drugs, consistent with the study by M.S.K et al. The total mean score of swallowing disorders was 17.4 points. This result is similar to that of a previous study by Kim et al. [29], that is, 17.1 points [29], which is judged as the mean swallowing disorder score for Korean older adults. Moreover, the mean BMI value was 23.5 kg/m^2^, which corresponds to overweight, similar to the study result of P.J et al. [30]. Among the participants, the proportion of obese was high at 31.7% on average, and the proportion of underweight was relatively low. MNA, an index for assessing nutritional status, is a simple, useful, and reliable tool for older adults. This indicator can be divided into normal, at risk of malnutrition, and malnutrition, and effective nutritional interventions can be obtained. More than half of our participants were at risk of malnutrition or already had malnutrition. In an international study by C and G et al. [31,32], approximately 61% of older adults aged 65 years or older were malnourished, similar to our study. However, our study revealed that the number of participants with malnutrition decreased in both groups after the intervention. In particular, a significant decrease in the intervention group suggests an effect of improvement after provision of nutrition education and senior-friendly diet. Senior-friendly excellent foods are customized products manufactured by adjusting the hardness, viscosity, shape, and ingredients of meals to facilitate older adults’ intake, nutrition, digestion, and absorption. Food intake starts with the teeth, followed by the gums and the tongue. Currently, 113 products have been selected as senior-friendly foods [33], and various aspects such as chewability level food groups should be developed in the future. According to the 2020 Nutrient Intake Standards for Koreans, the nutrient intake standards are aimed at healthy individuals and groups to maintain and improve their health, reduce the risk of chronic diseases related to diet, and ultimately improve their healthy lifespan. Energy is set at 1900 and 1500 Kcal for males and females [34], respectively, as well as 130 g of carbohydrates and 60 g of protein for males and 50 g of both for females. Compared with this criterion, the pre-energy intake in our study was 1360.7 Kcal in the intervention group and 1423.8 Kcal in the control group; regarding nutrients, 227.5 and 236.5 g of carbohydrates and 49.9 and 51.6 g of protein were consumed by the intervention and control groups, respectively. This result is similar to the results of previous studies [35,36] investigating vulnerable older adults with reduced mobility. However, as a result of applying the senior-friendly diet, energy and protein significantly improved in the intervention group. If the intake rate was set at 100% according to the Korean nutrient intake standard, in the intervention group, energy intake increased from 80% to 92.7%, protein exceeded 100%, and intake generally increased after the intervention period. However, the intake rate of vitamin A, vitamin D, vitamin C, niacin, calcium, and magnesium remained below 100%. Meanwhile, the intake of the control group was at a more vulnerable level. However, it is noteworthy that the intervention group had a significant increase in energy intake due to an increase in protein and fat intake, and a significant increase in sodium intake. The main foods of protein are meat, fish, tofu, eggs, etc., and many of the developed senior-friendly foods are composed of these foods as raw materials. As is known, these foods contain both protein and fat as major nutrients, so it is judged that the energy contribution rate for them has no choice but to go up. Excessive sodium intake can aggravate chronic diseases such as diabetes and hypertension, and inappropriate fat intake can lead to indigestion and an imbalance in nutrient intake. In order to meet the nutritional needs of the elderly, it is necessary to increase the amount of food, have a balanced diet, and supplement insufficient nutrients; however, on the contrary, monitoring is necessary to prevent inadequate nutritional intake. Senior-friendly foods used for meals are actually reflected in the nutrient intake; therefore, providing age-friendly foods to meals for older adults with difficulty eating should be implemented. According to the eSPPB analysis, the preliminary results of the two groups showed no difference, and the average score indicated frailty. The control group tended to have physical function decline over time; conversely, the frailty and total frailty rates of the intervention group significantly improved. The eSPPB tool, which has been verified to be reliable in various studies, can be measured in four ways: Robust, Prefrailty, Frailty, and Disability. Although eSPPB can be accurately and easily measured, installing it takes a long time, and evaluating older adults with limited activities can be difficult. Compared with previous studies such as that of H.W.J. [37], our participants were relatively more vulnerable, possibly because of the relatively high rate of frailty resulting from a high proportion of super-aged people in this study and the fact that they were receiving community care services. Community care provides housing, medical care, nursing care, and care services so that older adults can experience a healthy state while they age at home, thereby relieving widespread care anxiety and improving their quality of life in the face of a super-aged society. It is a policy that can be drastically improved. Actively supporting the vitalization of these policies and appropriate services for older adults is necessary. Furthermore, after the intervention, the total cholesterol, LDL-C, HDL-C, triglyceride, and hemoglobin levels were 143.4 mg/dL, 71.6 mg/dL, 48.8 mg/dL, 140.88 mg/dL, and 12.3 g/dL in the intervention group, respectively. Between groups, the indicator showing the most significant difference was the nutritional status evaluation score, followed by energy intake, protein intake, eSPPB score, grip strength, hemoglobin, and hematocrit. Most of the indicators in the control group tended to decrease or worsen over time, whereas nutritional and functional health status improved in the intervention group. Factors influencing the prevention of sarcopenia include exercise [38] and nutritional intake [39], and among dietary factors, energy [40,41], protein [42,43], and antioxidant nutrients [44] are especially important for sarcopenia and reported to be related. The recovery of energy is related to muscle mass over long periods of time, and the potential for energy distribution has been found to combine the electrical phases, despite of course not insignificant supplies. Therefore, there is a need to recover a fixed amount of energy and power to maintain adult gym muscle mass. The improvement in nutritional status or frailty indicators in this study was considered to be due to the complex application of an increase in diet intake, which was a problem, eventually leading to an increase in nutrients intake, and a management education program to maintain. In addition, education should be developed into multi-dimensional education programs such as nutrition management, frailty, chronic disease management, and oral hygiene. Providing a diet using senior-friendly excellent foods significantly improved frailty, nutritional status, nutrient intake, and blood tests. We also observed the effectiveness of various health indicators for older adults in improving and developing various systems for the expansion of the senior-friendly food industry in Korea. Many difficulties were encountered during the process of conducting research on a large number of older adults who were receiving government care. Special meals for older adults are absolutely necessary and must be adopted according to the circumstances of each country. The improvement of various health conditions when using senior-friendly foods leads to medical cost reduction. For instance, the decrease in blood glucose will result in saving KRW 610,000 per person annually and KRW 706.8 billion for older adults aged 65 years or older by our report. We believe that consuming a precise and complete diet by supplementing foods that can fill up the lack of nutrients is essential, considering that most of the foods are semiprepared, although senior-friendly foods should be easy to ingest. To this end, menus using senior-friendly foods should be developed, and active support systems such as linking with public institutions and public assistance should be prepared.

## 5. Conclusions

A senior-friendly food diet considering the chewing and swallowing function of older adults has a positive effect on the health of the aging population in terms of nutrients, and can greatly help improve the quality of life. Keeping a balanced nutritional status by maintaining a safe and steady dietary intake and preventing and managing frailty are necessary. Therefore, senior-friendly foods may be beneficial.

## Figures and Tables

**Table 1:** General characteristics of participants in the study.

Variables	Total *n* = 180	Intervention *n* = 154	Control *n* = 26	*p*-Value
Age (yrs)				
65–74	9 (5.0)	7 (4.5)	2 (7.7)	0.683
75–84	98 (54.4)	83 (53.9)	15 (57.7)	
85+	73 (40.6)	64 (41.6)	9 (34.6)	
Average (Mean ± S.D)	82.7 ± 5.9	82.7 ± 6.0	82.5 ± 5.7	0.873
Gender				
Men	67 (37.2)	60 (39.0)	7 (26.9)	0.279
Women	113 (62.8)	94 (61.0)	19 (73.1)	
Activity status				
Self	159 (88.3)	134 (87.0)	25 (96.2)	0.398
Assistant	19 (10.6)	18 (11.7)	1 (3.8)	
Impossible	2 (1.1)	2 (1.3)	0 (0.0)	
Family status				
Living alone	161 (89.4)	136 (88.3)	25 (96.2)	0.458
With spouse	15 (8.3)	14 (9.1)	1 (3.8)	
With family	4 (2.2)	4 (2.6)	0 (0.0)	
Disease history				
Cardiovascular	120 (66.7)	102 (66.2)	18 (69.2)	0.767
Endocrine	59 (32.8)	53 (34.4)	6 (23.1)	0.431
Musculoskeletal	77 (42.8)	62 (40.3)	15 (57.7)	0.178
Gastrointestinal	5 (2.8)	3 (1.9)	2 (7.7)	0.104
Three or more medications	145 (80.6)	126 (81.8)	19 (73.1)	0.630
Oral Health Impact Profile-14	3.3 ± 1.0	3.3 ± 1.1	3.3 ± 1.0	0.935
Dysphagia Handicap Index	17.4 ± 21.4	17.6 ± 21.8	16.0 ± 18.8	0.698
Body Mass Index (kg/m^2^)	23.5 ± 3.6	23.5 ± 3.6	23.4 ± 4.0	0.906
<18.5 (underweight)	15 (8.3)	13 (8.4)	2 (7.7)	0.564
18.5–22.9 (normal)	69 (38.3)	58 (37.7)	11 (42.3)	
23.0–24.9 (overweight)	39 (21.7)	35 (22.7)	4 (15.4)	
≥25 (obesity)	57 (31.7)	48 (31.2)	9 (34.6)	

Data were reported as mean ± standard deviation for continuous variable and *n* (%) for categorical variable. *p*-value was calculated by Student’s *t*-test or Mann–Whitney U test for continuous variable and chi-squared test or Fisher’s exact test for categorical variables as appropriate.

**Table 2:** Changes of nutritional status for each group.

Variables	Intervention *n* = 154			Control *n* = 26				
	Pre	Post	*p*-Value ^a^	Pre	Post	*p*-Value ^b^	*p*-Value ^c^	*p*-Value ^d^
Nutritional Status								
Total score	22.9 ± 3.7	23.6 ± 3.4	<0.001	23.5 ± 3.5	22.7 ± 3.7	0.004	0.452	0.184
Adequate	73 (47.4)	88 (57.1)	<0.001	12 (46.2)	13 (50.0)	<0.001	0.789	0.600
At risk of malnutrition	63 (40.9)	56 (36.4)		12 (46.2)	12 (46.2)			
Malnutrition	18 (11.7)	10 (6.5)		2 (7.7)	1 (3.8)			
Nutrients intake								
Energyr (kcal)	1360.7 ± 210.5	1576.7 ± 373.5	<0.001	1423.8 ± 185.3	1353.5 ± 344.1	0.257	0.153	0.005
Carbohydrater (g)	227.5 ± 40.2	231.5 ± 62.4	0.539	236.5 ± 38.5	191.8 ± 51.3	<0.001	0.289	0.003
Proteinr (g)	49.9 ± 14.9	60.7 ± 14.7	<0.001	51.6 ± 10.1	53.7 ± 13.0	0.346	0.579	0.023
Fatr (g)	26.4 ± 14.6	41.1 ± 12.8	<0.001	28.9 ± 13.6	38.8 ± 12.9	0.016	0.414	0.414
Fiberr (g)	22.2 ± 8.5	21.4 ± 7.0	0.411	22.4 ± 5.6	21.1 ± 7.1	0.439	0.907	0.838
Vitamin A (μg)	516.1 ± 352.0	433.6 ± 212.6	0.021	564.2 ± 269.4	458.1 ± 226.3	0.091	0.507	0.591
Vitamin B1 (mg)	1.3 ± 0.5	1.2 ± 0.4	0.289	1.5 ± 0.5	1.5 ± 0.5	0.998	0.089	0.018
Vitamin B2 (mg)	0.9 ± 0.4	1.1 ± 0.4	<0.001	1.0 ± 0.3	1.4 ± 0.5	0.007	0.045	0.021
Vitamin B6 (mg)	1.2 ± 0.8	1.5 ± 0.8	0.002	1.3 ± 0.3	3.1 ± 3.4	0.010	0.591	<0.001
Vitamin B12 (μg)	4.3 ± 4.7	5.7 ± 3.8	0.007	4.3 ± 3.3	5.8 ± 3.7	0.091	0.949	0.863
Vitamin C (mg)	50.0 ± 31.2	68.3 ± 33.7	<0.001	56.4 ± 26.4	72.6 ± 33.7	0.052	0.326	0.552
Niacin (mg)	10.6 ± 3.9	9.4 ± 3.2	0.003	12.2 ± 4.9	11.0 ± 3.7	0.293	0.074	0.029
Folic acid (mg)	381.1 ± 141.1	465.8 ± 186.7	<0.001	413.3 ± 118.8	475.1 ± 148.3	0.073	0.274	0.810
Vitamin D (μg)	1.6 ± 2.7	3.6 ± 3.5	<0.001	1.4 ± 1.6	4.9 ± 3.5	<0.001	0.687	0.088
Vitamin E (μg)	11.9 ± 5.3	14.0 ± 7.3	0.007	13.0 ± 5.2	17.1 ± 7.1	0.015	0.323	0.041
Vitamin K (μg)	187.1 ± 209.4	141.4 ± 153.3	0.026	195.4 ± 161.6	115.5 ± 127.7	0.041	0.847	0.415
Pantothenic acid (mg)	3.7 ± 1.0	3.4 ± 1.1	0.006	4.0 ± 1.1	3.8 ± 1.2	0.593	0.186	0.080
Calcium (mg)	377.3 ± 165.0	533.0 ± 238.1	<0.001	371.0 ± 119.9	426.5 ± 144.2	0.101	0.853	0.028
Phosphorus (mg)	843.2 ± 260.5	758.3 ± 241.2	0.003	903.0 ± 162.9	860.2 ± 239.1	0.366	0.259	0.047
Sodium (mg)	2973.0 ± 1306.8	4210.0 ± 1796.7	<0.001	3458.3 ± 1360.0	3962.2 ± 1256.2	0.097	0.083	0.500
Potassium (mg)	2192.8 ± 934.0	2175.8 ± 700.9	0.860	2386.3 ± 780.9	2422.1 ± 669.3	0.804	0.320	0.097
Iron (mg)	13.7 ± 5.2	14.3 ± 5.6	0.314	13.9 ± 3.1	13.6 ± 3.8	0.692	0.829	0.531
Magnessium (mg)	113.9 ± 60.6	73.3 ± 32.8	<0.001	129.8 ± 38.7	87.1 ± 32.2	<0.001	0.197	0.048
Zinc (mg)	8.6 ± 2.6	7.1 ± 2.2	<0.001	9.3 ± 1.7	9.0 ± 4.0	0.702	0.220	0.001
Copper (mg)	607.2 ± 351.6	369.6 ± 169.9	<0.001	696.0 ± 272.2	422.5 ± 165.5	<0.001	0.222	0.142
Selenium (mg)	37.4 ± 22.9	37.5 ± 22.7	0.969	47.9 ± 26.7	53.0 ± 22.9	0.495	0.037	0.002
Cholesterol (mg)	125.1 ± 145.9	320.1 ± 185.8	<0.001	156.8 ± 153.7	315.8 ± 217.2	0.014	0.311	0.915

Data were reported as mean ± standard deviation for continuous variable and *n* (%) for categorical variable. The *p*-value was calculated by paired *t*-test or Mann–Whitney U test for continuous variable and chi-squared test or Fisher’s exact test for categorical variables as appropriate. The *p*-value ^a^ was presented as pre-post differences in intervention group. The *p*-value ^b^ was presented as pre-post differences in control group. The *p*-value ^c^ was presented as pre-differences between two groups. *p*-Value ^d^ was presented as post-differences between two groups.

**Table 3:** Changes of functional capacity and clinical features for each group.

Variables	Intervention *n* = 154			Control *n* = 26				
	Pre	Post	*p*-Value ^a^	Pre	Post	*p*-Value ^b^	*p*-Value ^c^	*p*-Value ^d^
Electronic Short Physical Performance Battery	3.9 ± 2.2	4.4 ± 2.4	<0.001	4.1 ± 1.9	3.8 ± 1.8	0.050	0.767	0.221
Disability	75 (48.7)	58 (37.7)	<0.001	12 (46.2)	13 (50.0)	0.451	0.858	0.294
Frailty	61 (39.6)	70 (45.5)		12 (46.2)	10 (38.5)			
Prefrailty	16 (10.4)	22 (14.2)		2 (7.6)	3 (11.5)			
Robust	2 (1.3)	4 (2.6)		0 (0.0)	0 (0.0)			
Hand grip (kg)	14.7 ± 8.0	16.2 ± 6.4	<0.001	14.1 ± 7.8	13.1 ± 6.8	0.131	0.721	0.024
Quality of life (score)	0.7 ± 0.2	0.8 ± 0.2	<0.001	0.8 ± 0.1	0.8 ± 0.2	0.329	0.219	0.650
Systolic blood pressure (mmHg)	141.0 ± 20.5	140.6 ± 20.7	0.784	139.0 ± 27.9	137.1 ± 19.2	0.659	0.666	0.423
Diastolic blood pressure (mmHg)	76.3 ± 12.4	77.3 ± 12.3	0.323	76.9 ± 17.3	73.8 ± 14.1	0.141	0.823	0.183
Glucose (mg/dL)	144.0 ± 55.5	135.1 ± 40.3	0.009	138.5 ± 40.8	140.2 ± 40.5	0.661	0.635	0.368
C-reactive protein (mg/dL)	0.5 ± 0.1	0.7 ± 0.2	<0.001	0.5 ± 0.0	0.6 ± 0.1	<0.001	0.511	0.744
Total cholesterol (mg/dL)	153.8 ± 40.8	143.4 ± 34.8	<0.001	164.6 ± 35.5	157.3 ± 31.0	0.116	0.208	0.057
LDL cholesterol (mg/dL)	74.7 ± 34.6	71.6 ± 31.2	0.068	84.2 ± 28.9	79.8 ± 25.3	0.204	0.185	0.207
HDL cholesterol (mg/dL)	51.6 ± 14.0	48.8 ± 13.8	<0.001	50.5 ± 12.8	47.1 ± 15.3	0.072	0.707	0.564
Triglyceride (mg/dL)	154.4 ± 74.8	140.8 ± 65.1	0.003	163.0 ± 83.9	151.3 ± 55.2	0.264	0.594	0.435
Hemoglobin (g/dL)	12.4 ± 2.1	12.3 ± 1.8	0.634	12.4 ± 2.0	11.4 ± 2.1	<0.001	0.997	0.015
Hematocrit (%)	36.7 ± 6.4	36.7 ± 5.2	0.909	38.3 ± 4.5	34.4 ± 5.1	0.008	0.249	0.036

Data were reported as mean ± standard deviation for continuous variable and *n* (%) for categorical variable. *p*-value was calculated by paired *t*-test or Mann–Whitney U test for continuous variable and chi-squared test or Fisher’s exact test for categorical variables as appropriate. The *p*-Value ^a^ was presented as pre-post differences in intervention group. The *p*-Value ^b^ was presented as pre-post differences in control group. The *p*-Value ^c^ was presented as pre-differences between two groups. The *p*-Value ^d^ was presented as post-differences between two groups.

**Table 4:** Results of endpoints indicative.

Endpoints	Intervention	Control	Between-Group Difference (95% CI)	*p*-Value between Groups
Mini Nutritional Assessment	+0.7	−0.8	1.6 (1.0, 2.1)	*p* < 0.001
Energy intake (kcal)	+216.0	−70.2	286.2 (110.2, 462.2)	0.001
Protein intake (g)	+10.8	+2.1	8.7 (1.3, 16.2)	0.022
eSPPB (score)	+0.5	−0.3	0.7 (0.4, 1.1)	*p* < 0.001
Hand grip (kg)	+1.4	−1.1	2.5 (0.7, 4.3)	0.006
Quality of life (score)	+0.1	+0.0	0.1 (−0.0, 0.2)	0.052
Systolic blood pressure (mmHg)	−0.4	−1.8	1.5 (−6.2, 9.2)	0.709
Diastolic blood pressure (mmHg)	+1.1	−3.0	4.1 (−1.1, 9.4)	0.125
Total cholesterol (mg/dL)	−10.4	−7.2	3.2 (−13.0, 6.7)	0.527
LDL cholesterol (mg/dL)	−3.1	−4.5	1.4 (−7.2, 9.9)	0.757
HDL cholesterol (mg/dL)	−2.8	−3.4	0.6 (−3.2, 4.4)	0.749
Triglyceride (mg/dL)	−13.6	−11.7	2.0 (−24.6, 20.7)	0.865
Hemoglobin (g/dL)	−0.1	−1.0	1.0 (0.3, 1.6)	0.003
Hematocrit (%)	−0.1	−3.9	3.8 (0.8, 6.9)	0.013

Data are expressed as mean (95% confidence interval). All data were derived from a linear mixed-effects model. For each group, data are expressed as change from baseline to post-intervention, determined by the time coefficients (95% confidence interval) of the model. Between-group differences were determined with time x group interaction. A plus sign indicates an increasing value, and a minus sign indicates a decreasing value.

## Data Availability

The data that support the findings of this study are available from the corresponding author upon reasonable request.
